# A two-dimensional spin field-effect switch

**DOI:** 10.1038/ncomms13372

**Published:** 2016-11-11

**Authors:** Wenjing Yan, Oihana Txoperena, Roger Llopis, Hanan Dery, Luis E. Hueso, Fèlix Casanova

**Affiliations:** 1 CIC nanoGUNE, Tolosa Hiribidea 76, Donostia-San Sebastian, 20018 Basque Country, Spain; 2 Department of Electrical and Computer Engineering, University of Rochester, Rochester, New York 14627, USA; 3 Department of Physics and Astronomy, University of Rochester, Rochester, New York 14627, USA; 4 IKERBASQUE, Basque Foundation for Science, Bilbao, 48013 Basque Country, Spain

## Abstract

Future development in spintronic devices will require an advanced control of spin currents, for example by an electric field. Here we demonstrate an approach that differs from previous proposals such as the Datta and Das modulator, and that is based on a van de Waals heterostructure of atomically thin graphene and semiconducting MoS_2_. Our device combines the superior spin transport properties of graphene with the strong spin–orbit coupling of MoS_2_ and allows switching of the spin current in the graphene channel between ON and OFF states by tuning the spin absorption into the MoS_2_ with a gate electrode. Our proposal holds potential for technologically relevant applications such as search engines or pattern recognition circuits, and opens possibilities towards electrical injection of spins into transition metal dichalcogenides and alike materials.

## Abstract (web summary)

By forming heterostructures of different layered two-dimensional materials, functional spintronic devices may be built by exploiting the materials' different spin-orbit coupling and spin transport properties. Here, the authors demonstrate a spin switch in a gated structure of graphene and MoS_2_.

## Graphical abstract

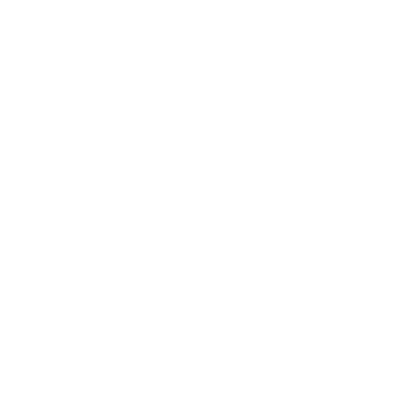

The integration of the spin degree of freedom in charge-based electronic devices has revolutionized both sensing and memory capability in microelectronics^1^. However, for allowing further development and a successful implementation of spin logic circuits, an electrical manipulation of spin currents is required. The approach followed so far, inspired by the seminal proposal of the Datta and Das spin modulator^2^, has relied on the spin–orbit field as a medium for electrical control of the spin current^3^,^4^,^5^,^6^. However, a challenge is to engineer a material that is capable of transporting spins over long distances and meanwhile has a strong enough spin–orbit coupling (SOC) to allow their electrical manipulation at temperatures above few Kelvin.

For example, carbon-based materials with intrinsic weak SOC, such as organic semiconductors^7^, carbon nanotubes^8^ and graphene^9^, have made a notable impact in spintronics. In particular, graphene has been proved to be ideal for long-distance spin transport (in excess of several micrometres)^10^,^11^,^12^,^13^,^14^,^15^. However, owing to its weak SOC, spin manipulation in this material has been mainly achieved by an external magnetic field through Hanle precession^10^,^13^,^14^. Although various approaches have been taken to enhance the SOC of graphene, for example through proximity effect^16^,^17^,^18^ or by atomic doping^19^, a direct evidence on the modulation of spin transport by an electric field remains elusive.

Meanwhile, transition metal dichalcogenides (TMDs) have emerged to complement graphene due to their unique optical, spin and valley properties^20^,^21^. Specifically, MoS_2_, the best-known member of that class, has a crossover from an indirect to a direct-gap semiconductor when thinned down to a monolayer (ML)^22^. Its electronic properties can be strongly modulated by gate, large current ON/OFF ratio as much as 1 × 10^8^ in ML and 1 × 10^6^ in multilayers have been found^23^,^24^. Its stronger SOC compared with that of graphene, arising from the *d-*orbitals of the transition metal atoms, offers new possibilities to employ the spin and valley degrees of freedom in TMDs^20^,^25^,^26^,^27^.

In our work, the combination of graphene with MoS_2_ in a heterostructure through weak van der Waals (vdW) forces^28^ allows us to engineer an alternative type of field-effect switch for spin transport. A spin current in the graphene section of the device is electrically injected from a ferromagnetic source terminal. The gate electrode controls how much of that spin current is absorbed by the intersecting MoS_2_ layer (spin sink) before its arrival to the ferromagnetic drain terminal. By tuning the gate voltage, we were able to switch the spin current between binary ON and OFF states at temperatures up to 200 K. The current device could be scalable and operative at room temperature, considering both the rapid progress made in chemical vapour deposition of two-dimensional (2D) materials and the theoretical performance of the materials involved.

## Results

### Device structure and measurement configurations

**Figure 1:**
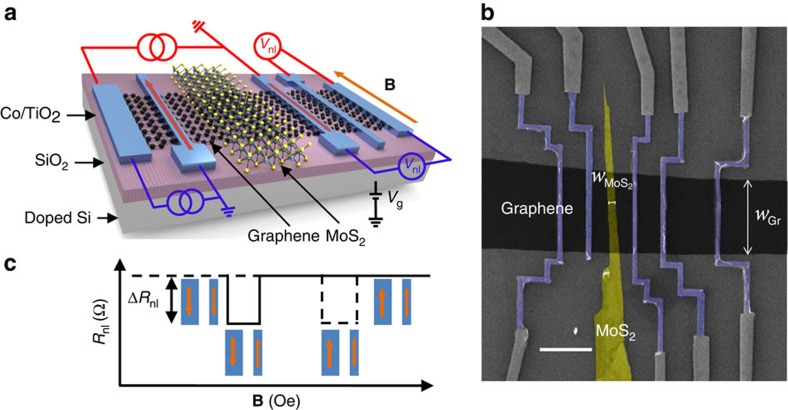
Illustration of the experiment and scanning electron microscope (SEM) image of the device. (**a**) Sketch of the 2D vdW heterostructure to be used for switching the spin transport. For the non-local measurement, a DC current (10 μA) is injected into graphene from a ferromagnetic Co electrode across a TiO_2_ barrier and a non-local voltage (*V*_nl_) is measured by a second Co electrode while sweeping the magnetic field **B**. The red- and blue-coloured circuit diagrams represent the measurement configurations in the reference graphene LSV (without MoS_2_ on top) and the graphene/MoS_2_ LSV (with MoS_2_ intercepting the spin current path). In the latter case, the spin current flowing in the graphene can be switched ON and OFF by modulating the conductivity of MoS_2_ using an electric field across a SiO_2_ dielectric (also shown in the diagram). (**b**) False-coloured SEM image of the LSV devices. The width of the graphene and MoS_2_ are 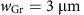 and 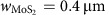, respectively. Scale bar, 2 μm. (**c**) An illustration of a typical non-local magnetoresistance measurement, where the non-local resistance *R*_nl_ switches between *R*_P_ and *R*_AP_ for parallel and antiparallel magnetization orientations of the Co electrodes. The spin signal is tagged as Δ*R*_nl_=*R*_p_−*R*_ap_.

A sketch of the 2D vdW heterostructure and the electrical measurement scheme is shown in Fig. 1a, whereas a scanning electron microscope image of the device is shown in Fig. 1b. Graphene flakes are exfoliated onto a highly doped Si substrate covered by 300 nm of SiO_2_. An ML graphene flake is identified according to its optical contrast^29^ and, subsequently, a few-layer MoS_2_ flake is transferred above it by all-dry viscoelastic stamping^30^. Several Co/TiO_2_ electrodes are patterned by electron-beam lithography and evaporated onto the graphene channel, to create lateral spin valves (LSVs; see Methods), which enable the injection and detection of pure spin currents in graphene in a non-local geometry^10^,^14^. The non-local resistance *R*_nl_=*V*_nl_*/I*, which depends on the relative orientation of the magnetisation of the injecting and detecting Co electrodes, is measured while sweeping the magnetic field **B** in-plane along the easy axis of the electrodes (see Fig. 1a for a sketch of the experimental geometry). Specifically, when the configuration of the magnetizations changes from parallel to antiparallel, *R*_nl_ switches from high (*R*_p_) to low (*R*_ap_) value. The spin signal is proportional to the amount of spin current reaching the detector, measured by Δ*R*_nl_=*R*_p_−*R*_ap_ (Fig. 1c).

### Spin transport in a reference graphene lateral spin valve

**Figure 2:**
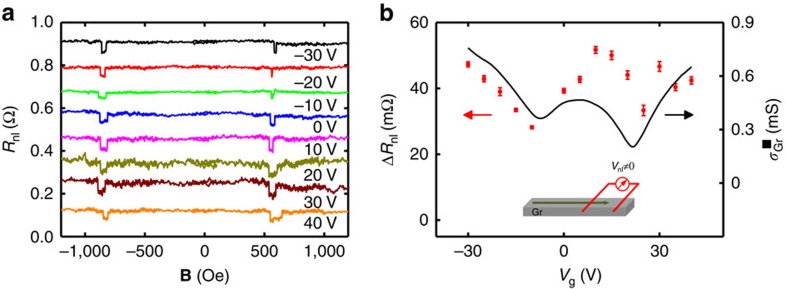
Spin transport in a reference graphene lateral spin valve. Measurements are done using the red-coloured circuit diagram in Fig. 1a. (**a**) Non-local resistance *R*_nl_ as a function of the magnetic field **B** measured at different *V*_g_ at 50 K. The current bias is 10 μA and the centre-to-centre distance between ferromagnetic electrodes (*L*) is 1 μm. Individual sweeps are offset in *R*_nl_ for clarity. (**b**) Spin signal Δ*R*_nl_ measured at different *V*_g_ (red circles). The black solid line shows the sheet conductivity of the graphene as a function of *V*_g_. The inset shows schematically the spin current (green arrow) reaching the detector in the full range of *V*_g_. Error bars are calculated using the s.e. associated with the statistical average of the non-local resistance in the parallel and antiparallel states.

We first study the spin transport in a graphene LSV without MoS_2_ (reference LSV). Figure 2a shows the measured *R*_nl_ as a function of **B** for different gate voltages (*V*_g_). On application of *V*_g_, the magnitude of the spin signal weakly varies, following the modification of the graphene sheet conductivity (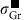) with *V*_g_, as can be observed in Fig. 2b. The correlation between Δ*R*_nl_ and 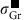 is a signature of a transparent interface between the Co/TiO_2_ electrodes and the graphene (∼250 Ω), as it is well established in the literature^14^.

### Spin transport in a graphene/MoS_2_ spin field-effect switch

**Figure 3:**
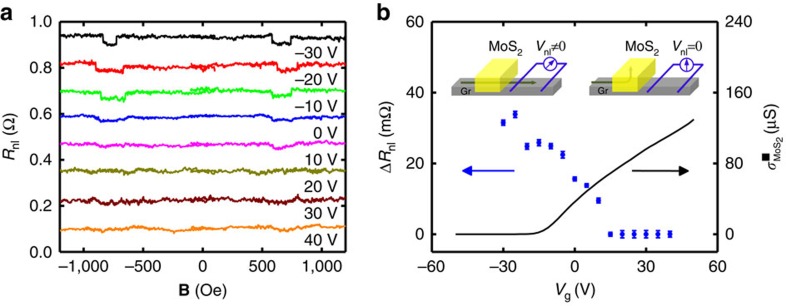
Spin transport in a graphene/MoS_2_ lateral spin valve. Measurements are done using the blue-coloured circuit diagram in Fig. 1a. (**a**) Non-local resistance *R*_nl_ measured as a function of the magnetic field **B** at different *V*_g_ at 50 K using 10 μA current bias and for a centre-to-centre distance between ferromagnetic electrodes (*L*) of 1.8 μm. Individual sweeps are offset in *R*_nl_ for clarity. (**b**) Gate modulation of the spin signal Δ*R*_nl_ (blue circles). The black solid line is the sheet conductivity of the MoS_2_ as a function of *V*_g_. The insets show schematically the spin current path (green arrow) in the OFF state (left inset) and the ON state (right inset) of MoS_2_. Error bars are calculated using the s.e. associated with the statistical average of the nonlocal resistance in the parallel and antiparallel states.

Next, we introduce the central results of our manuscript: the demonstration of spin switching by a gate voltage in a graphene/MoS_2_ LSV. Figure 3a shows *R*_nl_ of this device, while sweeping **B** for different values of *V*_g_, where a gradual decrease of the spin signal Δ*R*_nl_ with *V*_g_ can be observed. This behaviour is clearly seen in Fig. 3b, where Δ*R*_nl_ is plotted as a function of *V*_g_, showing the decay of Δ*R*_nl_ towards zero at positive values of *V*_g_, in contrast with the weakly varying spin signal measured in the reference LSV (see Fig. 2b). Figure 3b also plots the MoS_2_ sheet conductivity (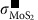) from a reference device revealing an opposite gate voltage dependence to that of Δ*R*_nl_. For large negative *V*_g_, the semiconducting MoS_2_ is in the low conductivity OFF state and the measured Δ*R*_nl_ value is comparable to that of the reference LSV, reaching the ON state of the device. This result is expected, considering that the electrode spacing here is slightly longer than in the reference LSV (1.8 versus 1 μm; see Fig. 1b for comparison). Sweeping the gate voltage towards positive values brings the MoS_2_ towards its high conductivity ON state, where 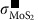 increases by more than six orders of magnitude compared with the OFF state. Simultaneously, the spin current reaching the detector and the corresponding Δ*R*_nl_ gradually decrease towards zero (see Fig. 3b), reaching the OFF state of the device for *V*_g_>15 V. The change in spin signal per gate voltage unit in our device is ∼0.7 mΩ V^−1^. The results are completely reproducible upon multiple gate voltage sweeps and temperature cycles, evidencing the robustness of the effect (Supplementary Fig. 1). Similar results to those in Fig. 3b are also observed at temperatures up to 200 K (Supplementary Fig. 2).

This control of the spin current directly demonstrates the 2D spin field-effect switch. This proof-of-principle effect can be enhanced by using a different dielectric, for example, layered hexagonal BN^24^,^31^, or by tuning the interface resistance between graphene and Co^14^.

## Discussion

The switching of spin transport using the graphene/MoS_2_ vdW heterostructure relies on the absorption of spins travelling through the graphene by the MoS_2_, as schematically illustrated in the inset of Fig. 3b. To support this argument, we make use of the spin resistances of the channel (graphene) and the absorbing material (MoS_2_), which are the main control parameters in the spin absorption mechanism. Roughly, they can quantify how easily the spin current flows through each of the materials, in the same way in which one can estimate a charge current flow in parallel electrical resistors. The spin resistances of graphene and MoS_2_ can be expressed as 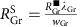 and 
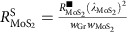
, respectively (Supplementary Note 2); where 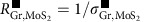 are their sheet resistances, 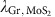 their spin diffusion lengths and 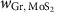 their widths (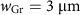 and 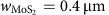). We have estimated the intrinsic spin lifetime in bulk MoS_2_ to be in the range of 10 ps (see Supplementary Note 2). For this estimation, we have considered electron interaction with flexural phonons and found weak temperature dependence of the spin relaxation in accord with our experimental results and in contrast to the findings in ML MoS_2_ (ref. 27). The lack of space inversion symmetry in a ML has two effects on the spin transport. The first one is to increase the amplitude of the spin-flip matrix element^26^. The second effect is to induce spin splitting of the energy bands at the *K* point. Although the former effect enhances spin relaxation, the latter one suppresses it when the spin splitting is large enough to exclude elastic scattering. In *p*-type ML TMDs, for example, the spin splitting in the valence band is of the order of hundreds of meV and the overall spin lifetime is prolonged compared with bulk. In this view, the spin degeneracy of the energy bands in few-layer TMDs renders these materials ideal spin sinks when put in proximity to graphene. Using the estimated spin lifetime in bulk MoS_2_, we calculate 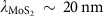 in the OFF state of the device at *V*_g_=40 V. In contrast, the spin diffusion length in graphene is much longer, being 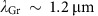 estimated from Hanle measurements on a reference device (Supplementary Fig. 3). Substituting the spin diffusion lengths, the electrical properties and geometrical factors of graphene and MoS_2_, we obtain 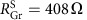 and 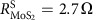 *V*_g_=40 V (Supplementary Note 2). The fact that 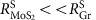 demonstrates the capability of the MoS_2_ to absorb spins from the graphene channel. This is further supported by the low graphene/MoS_2_ barrier height at high positive *V*_g_ (ref. 32), making the interface resistance sufficiently low for efficient spin absorption.

The situation completely changes when the gate voltage *V*_g_ is swept towards negative values. At *V*_g_=−30 V, the MoS_2_ conductivity 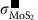 decreases by more than six orders of magnitude from *V*_g_=40 V, which leads to a similar increase in 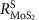. Therefore, we have 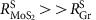, preventing spin absorption by MoS_2_. Although the very large spin resistance of MoS_2_ alone is sufficient to support the vanishing spin absorption, we note that the interface resistance between graphene and MoS_2_ also increases^32^, further preventing probable spin absorption into the MoS_2_. Therefore, the gate dependence of the graphene/MoS_2_ interface resistance acts as a positive feedback, further improving the performance in the regime between the fully ON and OFF state of the device.

The inverse correlation between the spin signal Δ*R*_nl_ and the MoS_2_ conductivity 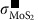 can be clearly seen in Fig. 3b. This correlation supports the aforementioned argument and discards other scenarios, such as spin dephasing in possible trap states at the graphene/MoS_2_ interface. The fact that similar results to those in Fig. 3b are also observed at 200 K indicates that the effect barely changes with temperature and therefore is incompatible with the exponential temperature dependence expected for capture and escape in trap states (Supplementary Note 1). Next, we confirm the spin absorption mechanism by computing the expected spin signal ratio, 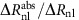, which quantifies the relative amount of spins deviating from the graphene channel towards the MoS_2_ (ref. 33):


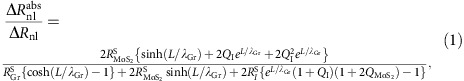


where 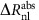 and Δ*R*_nl_ are the spin signals with and without spin absorption by the MoS_2_; 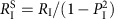 is the spin resistance of the Co/TiO_2_/graphene interface, *R*_I_ is the interface resistance and *P*_I_ is the interface spin polarisation; and finally, 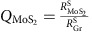 and 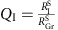. Assuming the interface is transparent at 40 V, one can calculate the expected spin signal ratio when the MoS_2_ is fully ON. Substituting the known parameters into equation (1), one gets 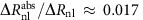 at *V*_g_=40 V (Supplementary Note 3). The very small value calculated for 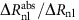 predicts a strong spin absorption, which confirms this scenario to be responsible for the observed experimental results of Fig. 3b.

Compared with previous Datta and Das-like spin modulators^3^,^4^,^5^,^6^, the electrical manipulation of spin transport in our 2D spin field-effect switch is observed at much higher temperature (up to 200 K versus few or sub K). It also displays well-defined ON and OFF states, which are easily controlled by the gate electric field instead of an oscillatory spin signal. Moreover, there is plenty of room for the optimization of the device performance. For instance, by incorporating tunnel barriers with higher resistance, the spin signal and thus the difference between the ON and OFF states could be increased by two orders of magnitude^34^,^35^,^36^. The threshold voltage required to turn ON and OFF the device can be reduced by replacing SiO_2_ with a thinner dielectric of larger dielectric constant, such as HfO_2_ (ref. 37) or hexagonal BN^24^,^31^. With the above improvements to the fabrication process, and considering the robust performance of MoS_2_ transistors at room temperature^23^,^24^, a room-temperature 2D spin field-effect switch is envisioned. The recent advances in chemical growth of high quality 2D layered materials^21^,^38^ and their heterostructure multilayers^39^,^40^,^41^, as well as in homostructural^42^, and heterostructural^36^ tunnel barriers for spin injection, may well lead to large-scale integration of the current device architecture. Aside from the potential technological applications, the spin absorption effect in our experiments provides a solution to electrically inject spins into 2D semiconducting TMDs, which has so far been elusive due to the conductivity mismatch problem^43^,^44^,^45^.

In conclusion, the seamless integration of two 2D layered materials with remarkably different spin–orbit coupling amplitudes leads to a device capable of both transporting and electrically controlling a spin current. The demonstrated 2D spin field-effect switch can improve the performance of search engines or pattern recognition circuits, wherein a large number of independent logic operations are executed in parallel^15^,^46^,^47^. Furthermore, the vdW heterostructure at the core of our experiments opens the path for fundamental research of exotic transport properties predicted for TMDs^20^,^25^,^26^.

## Methods

### Device fabrication

Fabrication of ML graphene samples uses the mechanical exfoliation method initiated in ref. 29. We first exfoliate bulk graphitic crystals onto a Nitto tape (Nitto SPV 224P) and repeat the cleavage process between three and five times until thin flakes can be identified visually by the eye. The Nitto tape with relatively thin flakes is pressed against a preheated Si substrate with 300 nm SiO_2_. After peeling off the Nitto tape, the substrate is examined under an optical microscope and ML graphene is identified by well-established optical contrast. We then prepare the MoS_2_/poly-dimethyl siloxane stamp following ref. 30. First, a MoS_2_ crystal is exfoliated twice using the Nitto tape and transferred on to a piece of poly-dimethyl siloxane (Gelpak PF GEL film WF × 4, 17 mil.). After identifying the desired few-layer MoS_2_ flake using optical contrast, it is transferred on top of graphene after slowly removing the viscoelastic stamp.

The lateral spin valve is formed following a standard nanofabrication procedure including electron-beam lithography, metal deposition and metal lift-off in acetone. 5 Å of Ti are deposited by electron-beam evaporation and left to oxidize in air for 0.5 h before depositing 35 nm of Co using electron-beam evaporation.

### Electrical measurements

The measurements are performed in a Physical Property Measurement System by Quantum Design, using a ‘DC reversal' technique with a Keithley 2182, nanovoltmeter and a 6221 current source. A current bias of 10 μA is used unless stated in the text. Gate voltage is applied using a Keithley model 2636. The gate voltage is applied between the back of the doped Si substrate and the grounding electrode. As graphene layer on the bottom is ML, it does not fully screen the gate electric field. Therefore, the gate voltage modulates the charge carrier density in both graphene and the MoS_2._

### Data availability

All relevant data are available from the authors.

## Additional information

**How to cite this article:** Yan, W. *et al*. A two-dimensional spin field-effect switch. *Nat. Commun.* **7**, 13372 doi: 10.1038/ncomms13372 (2016).

**Publisher's note:** Springer Nature remains neutral with regard to jurisdictional claims in published maps and institutional affiliations.

## Supplementary Material

Supplementary Information Supplementary Figures 1-4, Supplementary Notes 1-3 and Supplementary References
