# Supplementary material for: A two-dimensional spin field-effect switch
Source: Nat Commun. 2016 Nov 11;7:13372. doi: 10.1038/ncomms13372 (PMC5114593; doi:10.1038/ncomms13372)
Supplement: Supplementary Information — Supplementary Figures 1-4, Supplementary Notes 1-3 and Supplementary References [file ncomms13372-s1.pdf]

Supplementary Note 1

A 2D spin field-effect switch

The spin switching effect is robust and reproducible upon multiple gate sweeps and temperature cycles. Supplementary Fig. 1 shows non-local resistance measurements at different gate voltages performed before the ones shown in the main text (Fig. 2 and 3) in a different cryostat. Furthermore, to rule out charging effects as the origin of the spin transistor effect when sweeping the gate voltage, the measurements were performed in a random order: -30 V, 50 V, 0 V, 20 V, -10 V and 10V. Same to that observed in Fig. 3 of the main text, at large positive gate voltage the spin transport channel is completely turned OFF and no spin signal is measured, confirming the robustness and reproducibility of the effect.

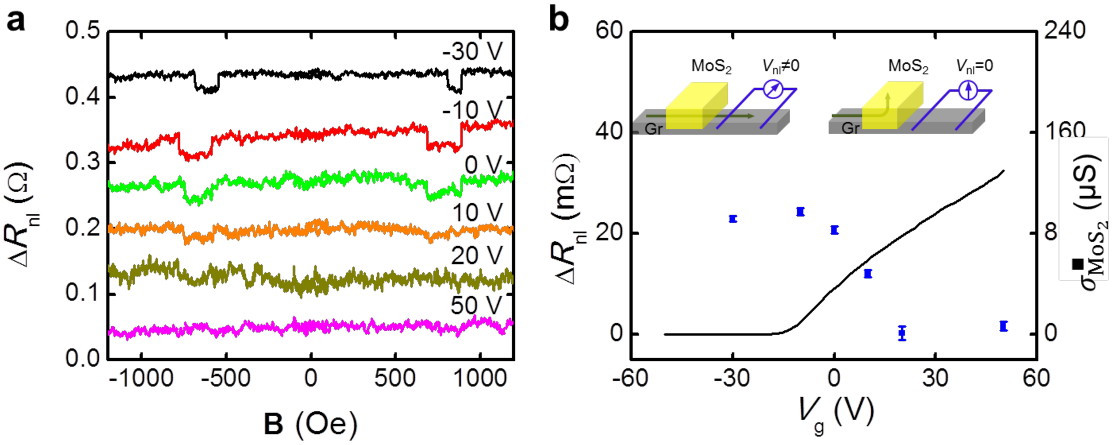

Supplementary Figure 1. Spin transport in the graphene/MoS_2 lateral spin valve at 50 K. (**a**), Non-local resistance R_{nl} measured at different V_g at 50 K using 10 μA current bias and for a centre-to-centre distance between ferromagnetic electrodes (L) of 1.8 μm. Individual sweeps are

offset in R_{nl} for clarity. (**b**), Gate modulation of the spin signal ΔR_{nl} (blue circles). The black solid line is the sheet conductivity of the MoS_2 as a function of V_g. The insets show schematically the spin current path (green arrow) in the off state (left inset) and the on state (right inset) of MoS_2. The measurements were performed in a different cryostat to the one used in the main text and taken in the following order of gate voltage: -30 V, 50 V, 0 V, 20 V, -10 V, 10 V, confirming the robustness and reproducibility of the effect.

Supplementary Figure 2 shows the spin transport measurements at 200 K for the devices shown in the main text. The spin signal of the reference graphene LSV at 200 K is similar to that at 50 K (compare Supplementary Fig. 2a and Fig. 2a in the main text), as expected from the results in Supplementary Fig. 3a and the literature[1,2], thus providing a fundamental building block on which room temperature spin switch can be built.

The comparison between Supplementary Fig. 2a and Supplementary Fig. 2b demonstrates the operation of the 2D spin switch at 200 K. While $\Delta \boldsymbol{R}_{\mathbf{nl}}$ in the reference graphene LSV varies slightly with V_g (Suppelmentary Fig. 2a), gate modulation of spin signal between ON and OFF states is clearly observed in the graphene/MoS_2 LSV (Supplementary Fig. 2b), evidencing that the spin absorption of MoS_2 does not depend on the temperature. This experimental fact rules out the scenario of spin dephasing in trap states at the graphene/MoS_2 interface, because it is incompatible with the exponential temperature dependence expected for capture and escape in trap states, and suggests a weak temperature dependence of the spin relaxation time of few-layer MoS_2, as predicted by our calculations (see Supplementary Note 2 below).

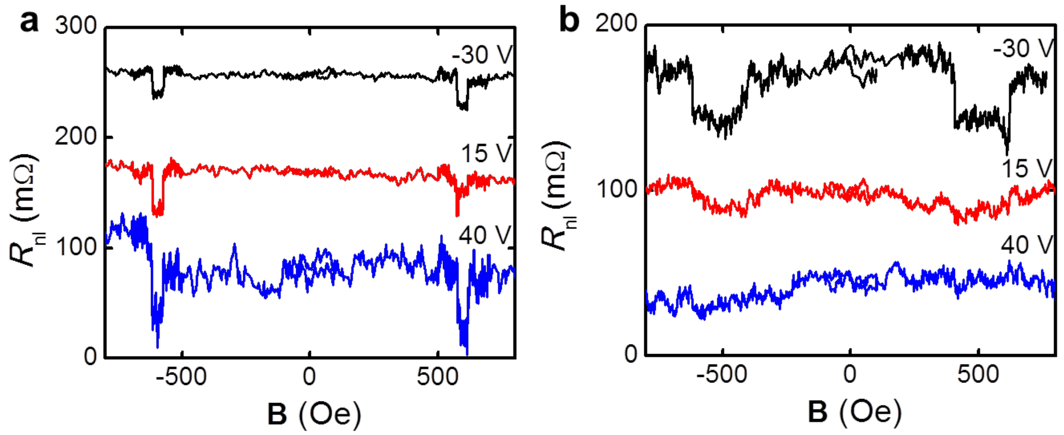

Supplementary Figure 2. Spin transport in reference graphene and graphene/MoS_2 lateral spin valves at 200 K. Non-local resistance R_{nl} measured at different V_g using 10 μA current bias and for (**a**) the reference graphene LSV, in which the centre-to-centre distance between ferromagnetic electrodes (L) is 1 μm. (**b**) the graphene/MoS_2 LSV, in which the centre-to-centre distance between ferromagnetic electrodes (L) is 1.8 μm. Individual sweeps are offset in R_{nl} for clarity.

Supplementary Note 2

Spin transport properties of graphene

The spin transport properties of graphene are studied in a typical graphene lateral spin valve as a function of temperature from 10 K to 300 K. The spin signal is weakly dependent on temperature, see Supplementary Fig. 3 and Ref. [1,2].

The spin relaxation in the graphene channel was studied using Hanle precession. The experiment is done by first setting the injecting and detecting Co/TiO_2 electrodes in the parallel or antiparallel magnetisation state along the length of the electrodes by applying an in-plane magnetic field **B**. The device is then rotated by 90° and the non-local resistance R_{nl} is measured while sweeping the magnetic field out-of-plane ($\mathbf{B}_\perp$). Supplementary Figure 3b shows the typical Hanle precession curve in the parallel (red

circles) and antiparallel (blue circles) magnetization configurations of the electrodes, after removing the background signal arising from the out-of-plane tilting of the electrodes magnetization at high fields by subtracting the antiparallel from the parallel data[3].

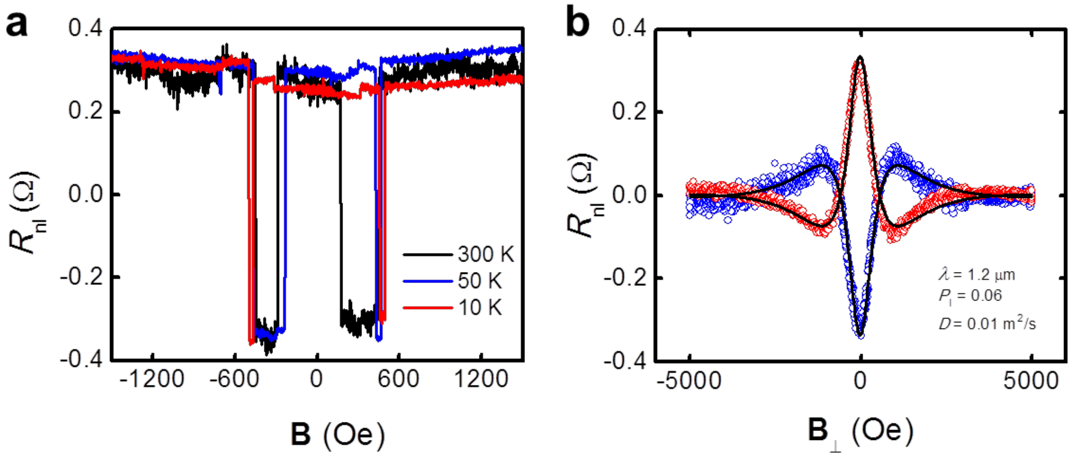

Supplementary Figure 3: Spin transport in a graphene lateral spin valve. (**a**) Non-local resistance R_{nl} measured with in-plane magnetic field sweep along the length of the electrodes at different temperatures. (**b**) Non-local resistance R_{nl} is measured under out-of-plane magnetic field $\mathbf{B}_\perp$ while the injecting and detecting Co/TiO_2 electrodes are parallel (red) and antiparallel (blue). The contribution from the out-of-plane rotation of the electrodes under $\mathbf{B}_\perp$ is removed by subtraction the antiparallel curve from the parallel curve. Spin diffusion length (λ), interface spin polarisation (P_I) and spin diffusion coefficient (D) are extracted by fitting Supplementary Equation 1 to the experimental data (black solid lines).

In order to fit the experimental data, we follow Ref. 4, which considers (i) spin precession and (ii) anisotropic spin absorption under the Co/TiO_2 injector contact. The following expression is used[4]:

$$R_{\mathrm{nl}} = -2R_{\mathrm{N}}\left(\frac{P_{\mathrm{F1}}}{1-P_{\mathrm{F1}}^2}\frac{R_{\mathrm{F1}}}{R_{\mathrm{N}}} + \frac{P_{\mathrm{I1}}}{1-P_{\mathrm{I1}}^2}\frac{R_{\mathrm{I1}}}{R_{\mathrm{N}}}\right)\left(\frac{P_{\mathrm{F2}}}{1-P_{\mathrm{F2}}^2}\frac{R_{\mathrm{F2}}}{R_{\mathrm{N}}} + \frac{P_{\mathrm{I2}}}{1-P_{\mathrm{I2}}^2}\frac{R_{\mathrm{I2}}}{R_{\mathrm{N}}}\right)\frac{C_{12}}{\det(\check{X})}, \quad (1)$$

where $R_{Fk} = \rho_F \lambda_F / A_{Ik}$ are the spin resistances of the kth FM contact (k =1 is the injector and k = 2 is the detector), with resistivity ρ_F, spin diffusion length λ_F and contact area of A_{Ik}; , $R_N = \frac{R_{Gr}^{\blacksquare} \lambda_{Gr}}{w_{Gr}}$ is the spin resistance of graphene calculated with its sheet resistance ($R_{Gr}^{\blacksquare}$), its spin diffusion length (λ_{Gr}) and width (w_{Gr}); $R_{Ik} = 1/G_{Ik}$ is the resistance of the kth interface, where $G_{Ik} = G_{Ik}^{\uparrow} + G_{Ik}^{\downarrow}$ is the conductance of the kth interface that considers both spin up and down channels; $P_{Ik} = \left(G_{Ik}^{\uparrow} - G_{Ik}^{\downarrow}\right)/\left(G_{Ik}^{\uparrow} + G_{Ik}^{\downarrow}\right)$ describes the interfacial spin polarization; and C_{12} and $\det(\check{X})$ are defined as[4]:

$$C_{12} = -\det \begin{pmatrix} \mathrm{Re}[\bar{\lambda}_\omega e^{-L/\bar{\lambda}_\omega}] & -\mathrm{Im}[\bar{\lambda}_\omega e^{-L/\bar{\lambda}_\omega}] & -\mathrm{Im}[\bar{\lambda}_\omega] \\ \mathrm{Im}[\bar{\lambda}_\omega] & r_{1\perp} + \mathrm{Re}[\bar{\lambda}_\omega] & \mathrm{Re}[\bar{\lambda}_\omega e^{-L/\bar{\lambda}_\omega}] \\ \mathrm{Im}[\bar{\lambda}_\omega e^{-L/\bar{\lambda}_\omega}] & \mathrm{Re}[\bar{\lambda}_\omega e^{-L/\bar{\lambda}_\omega}] & r_{2\perp} + \mathrm{Re}[\bar{\lambda}_\omega] \end{pmatrix}, \quad (2)$$

$$\check{X} = \begin{pmatrix} r_{1\parallel} + \mathrm{Re}[\bar{\lambda}_\omega] & \mathrm{Re}[\bar{\lambda}_\omega e^{-L/\bar{\lambda}_\omega}] & -\mathrm{Im}[\bar{\lambda}_\omega] & -\mathrm{Im}[\bar{\lambda}_\omega e^{-L/\bar{\lambda}_\omega}] \\ \mathrm{Re}[\bar{\lambda}_\omega e^{-L/\bar{\lambda}_\omega}] & r_{2\parallel} + \mathrm{Re}[\bar{\lambda}_\omega] & -\mathrm{Im}[\bar{\lambda}_\omega e^{-L/\bar{\lambda}_\omega}] & -\mathrm{Im}[\bar{\lambda}_\omega] \\ \mathrm{Im}[\bar{\lambda}_\omega] & \mathrm{Im}[\bar{\lambda}_\omega e^{-L/\bar{\lambda}_\omega}] & r_{1\perp} + \mathrm{Re}[\bar{\lambda}_\omega] & \mathrm{Re}[\bar{\lambda}_\omega e^{-L/\bar{\lambda}_\omega}] \\ \mathrm{Im}[\bar{\lambda}_\omega e^{-L/\bar{\lambda}_\omega}] & \mathrm{Im}[\bar{\lambda}_\omega] & \mathrm{Re}[\bar{\lambda}_\omega e^{-L/\bar{\lambda}_\omega}] & r_{2\perp} + \mathrm{Re}[\bar{\lambda}_\omega] \end{pmatrix}, \quad (3)$$

where $\bar{\lambda}_\omega = \tilde{\lambda}_\omega / \lambda_N$ with $\tilde{\lambda}_\omega = \lambda_N / \sqrt{i + i\omega_L \tau_{sf}}$ and the Larmor frequency $\omega_L = \gamma_e \mathbf{B}_\perp = \frac{g\mu_B}{\hbar} \mathbf{B}_\perp$; L is the distance between FM electrodes; $r_{k\parallel} = \left(\frac{2}{1-P_{Ik}^2} \frac{R_{Ik}}{R_N} + \frac{2}{1-P_{Fk}^2} \frac{R_{Fk}}{R_N}\right)$; and $r_{k\perp} = \frac{1}{R_N G_{Ik}^{\uparrow\downarrow}}$ with $G_{Ik}^{\uparrow\downarrow}$ being the spin mixing interface conductance.

For the fitting in Supplementary Fig. 3b, we assume the injecting and detecting electrodes have (i) the same spin polarisations ($P_{F1} = P_{F2} = P_F$ and $P_{I1} = P_{I1} = P_I$), (ii) the same interface resistances with the graphene channel, and following Ref. 4 (iii) an isotropic spin absorption, hence $G_{Ik}^{\uparrow\downarrow} = 1/(2R_{Ik} + 2R_{Fk})$. We fix the following experimental parameters: P_F = 0.12 (Ref. 5), $R_{I1} = R_{I2}$ = 10000 Ω, L = 2.26 μm, w_{Gr} = 0.73 μm, w_{F1} =

340 nm, w_{F2} = 230 nm, $R_{Gr}^{■} = 1317\ \Omega$, ρ_F = 19 μΩcm (Ref. 5), λ_F = 40 nm (Ref. [6,7]), $G_{I1}^{\uparrow\downarrow} = G_{I2}^{\uparrow\downarrow} = 5 \times 10^{-5}\ \Omega^{-1}$, and obtain P_I = 0.06, D = 0.01 m^2/s, λ_{Gr} = 1.2 μm.

Spin transport properties of MoS_2

We have made the following analysis and estimated the intrinsic spin relaxation time in bulk MoS_2 (τ_{MoS_2}) is between ~10 ps and ~30 ps at 50 K. The relaxation was calculated via interaction of electrons with flexural phonons, which are long wavelength out-of-plane undulations. These phonons are far more populated than in-plane acoustic phonons (sound waves) since the interlayer van der Waals (vdW) interactions render the out-of-plane long wavelength undulations to be nearly "resistance-free" compared with in-plane motion of atoms (since atoms are held by strong chemical bonds in the plane). As important, the flexural phonons are strongly coupled to spin-flips[8]. It is also important to note that the analysis ignores extrinsic spin relaxation due to interaction with impurities and, therefore, if the MoS_2 is impurity-rich the τ_{MoS_2} value can be somewhat smaller. In addition, we ignore intervalley spin-flip scattering between K and K' valleys due to time-reversal symmetry (which applies to both monolayer and bulk MoS_2)[8].

Due to the fact that vdW interactions lead to weak interlayer coupling, the scattering is essentially a two-dimensional problem; that is, the electron motion before and after the scattering are mainly in-plane. Next, we assume that flexural phonons obey a quadratic dispersion law as often found in unstrained vdW materials[9,10]. Specifically, $E_{ph} = \sqrt{\kappa\rho_m}q^2$, where E_{ph} is the flexural phonon energy, κ is the bending rigidity (~10 eV in MoS_2)[11], ρ_m is the area mass density (~$3 \cdot 10^{-7}$ gr/cm^2 in MoS_2)[12,13], and q is the phonon wavevector.

Next, we use symmetry arguments to estimate the spin-flip matrix element of electrons due to scattering with flexural phonons in bulk MoS_2. Due to space inversion, the wavevector dependence of the spin-flip matrix element is quadratic, $M_{\mathrm{bulk}} = D_{\mathrm{so}} q^2$, where D_{so} is a scattering constant (units of energy·cm) coming from the spin-orbit coupling part of the deformation potential. In monolayers, where space inversion symmetry is not respected, the spin-flip matrix element follows a linear relation with the phonon wavevector, $M_{\mathrm{mono}} = E_{\mathrm{so}} q$, where E_{so} is a spin-orbit coupling deformation potential, which for monolayer MoS_2 is $E_{\mathrm{so}} \sim 0.2$ eV[14]. Given the spin-orbit coupling is non-vanishing only in the vicinity of the atomic cores, E_{so} (monolayer) and D_{so} (bulk) are related by $D_{\mathrm{so}} \sim a\, E_{\mathrm{so}}$, where a is the lattice constant (~3 Å). Therefore, $D_{\mathrm{so}} \sim$ 0.6 eV·Å.

Using the dispersion of phonons and the spin-flip matrix element, the electron-phonon interaction that leads to intrinsic spin relation in the bulk is:

$$\left|< k,\uparrow \left|H_{\mathrm{electron-phonon}}\right| k+q,\downarrow >\right|^2 = \left[\frac{k_B T}{2A\rho_{\mathrm{m}}(\kappa/\rho_{\mathrm{m}})q^4}\right][D_{\mathrm{so}} q^2]^2 = \frac{k_B T (D_{\mathrm{so}})^2}{2A\kappa} \qquad (4)$$

where $k_{\mathrm{B}} T$ is the thermal energy, being k_{B} the Boltzmann constant and T the temperature, and A is the area of the flake. This area is cancelled when we turn the sum over all possible final scattered states into integral form. The interaction amplitude in the expression above is wavevector-independent due to cancelling effects between the phonon dispersion and spin-flip matrix element. This makes the summation over final

states trivial, where the Fermi golden rule yields the following spin relaxation rate:

$$\frac{1}{\tau_{\mathrm{MoS_2}}} = \frac{mk_{\mathrm{B}}T(D_{\mathrm{so}})^2}{\hbar^3\kappa}, \quad (5)$$

where m is the electron mass. In the case of bulk MoS_2, m is approximately the free electron mass. Plugging the numbers above, we get $\tau_{\mathrm{MoS_2}}\sim 30$ ps. As shown in the expression, the spin relaxation rate dependence on T is linear, being much weaker than the exponential dependence found in monolayers[15]. The reason is that the bands are spin-degenerate in bulk MoS_2. In monolayers, on the other hand, the bands are spin-split and since spin-flips are largely elastic, the top spin-split band should be populated to have a non-negligible spin-flip amplitude.

Next, we consider that the dispersion of flexural phonons is renormalized due to the coupling between bending and stretching degrees of freedom[9]. This coupling prevents violent undulations and crumpling. In this case, the dispersion of flexural phonons follows is $E_{\mathrm{ph}} = \sqrt[4]{k_{\mathrm{B}}T/\rho_{\mathrm{m}}}\sqrt{v_0}q^{3/2}$, where v_0 is the effective sound velocity ($5\cdot 10^5$ cm/s). Repeating the analysis above, a renormalized electron-phonon interaction that leads to spin flips in the bulk is

$$\left|< k,\uparrow \left|H_{\mathrm{renorm.electron-phonon}}\right|k+q,\downarrow>\right|^2 = \frac{q\sqrt{k_BT/\rho_m}(D_{\mathrm{so}})^2}{2Av_0}. \quad (6)$$

Since close to thermal equilibrium $q\sim\sqrt{2mk_{\mathrm{B}}T}/\hbar$, the summation over final states in the Fermi golden rule yields the following spin relaxation rate

$$\frac{1}{\tau_{\mathrm{MoS_2}}} = 4\pi \frac{mk_{\mathrm{B}}T(D_{\mathrm{so}})^2}{\hbar^4 v_0} \sqrt{\frac{2m}{\rho_m}}. \tag{7}$$

Therefore, the relaxation remains linear in *T*. Plugging numbers we get $\tau_{\mathrm{MoS_2}}$=10 ps.

Next, we convert $\tau_{\mathrm{MoS_2}}$ into $\lambda_{\mathrm{MoS_2}}$ by using the diffusion coefficient $D_{\mathrm{MoS_2}}$ as $\lambda_{\mathrm{MoS_2}} = \sqrt{D_{\mathrm{MoS_2}}\tau_{\mathrm{MoS_2}}}$. $D_{\mathrm{MoS_2}}$ depends on the mobility of the charge carriers in the MoS_2, $\mu_{\mathrm{MoS_2}}$, as $D_{\mathrm{MoS_2}} = \frac{\mu_{\mathrm{MoS_2}} k_B T}{\mathrm{e}}$.

In order to calculate $\mu_{\mathrm{MoS_2}}$, we use the $\sigma^{\blacksquare}_{\mathrm{MoS_2}}(V_{\mathrm{g}})$ data shown in Fig. 3b of the main text, which is representative of a typical MoS_2 flake transferred with the viscoelastic PDMS stamping (see methods) on a SiO_2 substrate. The results are reproducible from sample to sample because this transfer technique minimises the residues between the MoS_2 and the SiO_2 substrate and therefore enables an effective and repeatable field effect.

The $\sigma^{\blacksquare}_{\mathrm{MoS_2}}(V_{\mathrm{g}})$ data in Fig. 3b has been measured for a fixed source-drain voltage of $V_{\mathrm{SD}} = 0.26$ V and sweeping the gate voltage V_{g} while measuring the current passing between source and drain, I_{SD}. From this data, we can calculate the mobility $\mu_{\mathrm{MoS_2}}$ in the linear regime ($V_{\mathrm{g}} \gtrsim -15$ V)[16]: $\mu_{\mathrm{MoS_2}} = 84.6$ cm^2/(V·s). Therefore, $D_{\mathrm{MoS_2}} = 3.6 \cdot 10^{-5}$ m^2/s. Finally, we obtain $\lambda_{\mathrm{MoS_2}} \approx 20$ nm.

Spin resistances in two dimensional materials

Spin resistance is a term that describes the resistance spins experience in its volume of interaction *V*. The general expression for spin resistance R_{S} in any material is expressed as:

$$R_{\mathrm{S}} = \frac{\lambda^2 \rho}{V}$$

In the case of graphene, spin diffuses along a length of λ_{Gr} through a cross section area of $w_{\mathrm{Gr}} \times t_{\mathrm{Gr}}$.

$$R_{\mathrm{Gr}}^{\mathrm{S}} = \frac{\lambda_{\mathrm{Gr}}^{2} R_{\mathrm{Gr}}^{\blacksquare} t_{\mathrm{Gr}}}{\lambda_{\mathrm{Gr}} \times w_{\mathrm{Gr}} \times t_{\mathrm{Gr}}}$$

In the case of 2D material like graphene, the resistivity is normally expressed in terms of sheet resistance $R_{\mathrm{Gr}}^{\blacksquare}$ and $R_{\mathrm{Gr}}^{\blacksquare} = \frac{\rho}{t_{\mathrm{Gr}}}$. Therefore, one gets:

$$R_{\mathrm{Gr}}^{\mathrm{S}} = \frac{\lambda_{\mathrm{Gr}}^{2} R_{\mathrm{Gr}}^{\blacksquare} t_{\mathrm{Gr}}}{\lambda_{\mathrm{Gr}} \times w_{\mathrm{Gr}} \times t_{\mathrm{Gr}}} = \frac{R_{\mathrm{Gr}}^{\blacksquare} \lambda_{\mathrm{Gr}}}{w_{\mathrm{Gr}}} \quad (8)$$

In the case of spin absorption by a thick MoS_2 flake, the spin diffuses along the thickness of MoS_2 through a cross section area of $w_{\mathrm{Gr}} w_{\mathrm{MoS_2}}$. Its spin resistance is defined as the following equation[17]:

$$R_{\mathrm{MoS_2}}^{\mathrm{S}} = \frac{\rho_{\mathrm{MoS_2}} \lambda_{\mathrm{MoS_2}}^{2}}{w_{\mathrm{Gr}} w_{\mathrm{MoS_2}} \lambda_{\mathrm{MoS_2}} \tanh(t_{\mathrm{MoS_2}}/\lambda_{\mathrm{MoS_2}})}, \quad (9)$$

where $\tanh(t_{\mathrm{MoS_2}}/\lambda_{\mathrm{MoS_2}})$ terms is a geometrical factor that considers the length scale of the thickness of MoS_2 and $\lambda_{\mathrm{MoS_2}}$. This hyperbolic tangent term comes from the boundary condition where the spin current $I_{\mathrm{S}} = 0$ at the substrate, as detailed in Ref. 17.

In the case where the $\lambda_{\mathrm{MoS_2}} \gg t_{\mathrm{MoS_2}}$, the above equation reduces to:

$$R_{\mathrm{MoS_2}}^{\mathrm{S}} \approx \frac{R_{\mathrm{MoS2}}^{\blacksquare} (\lambda_{\mathrm{MoS_2}})^{2}}{w_{\mathrm{Gr}} w_{\mathrm{MoS_2}}} \quad (10)$$

where $R_{\mathrm{MoS_2}}^{\blacksquare} = 1/\sigma_{\mathrm{MoS_2}}^{\blacksquare}$ is the sheet resistance of MoS_2 which results in $R_{\mathrm{MoS_2}}^{\mathrm{S}} \approx 2.7\ \Omega$. However, considering in our case $\lambda_{\mathrm{MoS_2}}$ is of the order of the thickness of MoS_2 (20 nm and 7 nm, respectively, see previous sessions and Supplementary Fig. 4), one can get a more accurate estimation for $R_{\mathrm{MoS_2}}^{\mathrm{S}}$ by taking into account the length scale of the thickness of MoS_2 and $\lambda_{\mathrm{MoS_2}}$ using Supplementary Equation 9. The resistivity of MoS_2 is

$\rho_{\mathrm{MoS_2}} = t_{\mathrm{MoS_2}}/\sigma^{\blacksquare}_{\mathrm{MoS_2}} = 6.4 \cdot 10^{-5}\ \Omega \cdot \mathrm{m}$ is the resistivity of MoS_2. Eq. S9 results in $R^{\mathrm{S}}_{\mathrm{MoS_2}} = 2.8\ \Omega$. Because Supplementary Equation 9 and 10 result in very similar values, we consider the simplified expression of Supplementary Equation 10 for the rough analysis in this work.

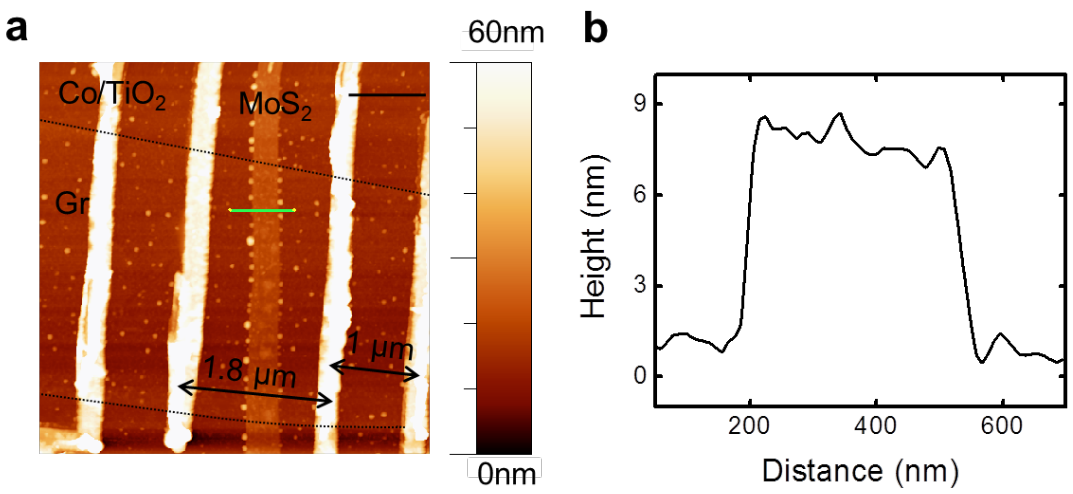

Supplementary Figure 4: Atomic force microscopy measurement on the devices. (**a**) Area scan showing the topography of the device, where graphene edge is traced by black dotted line and it is approximately 3 μm wide. The MoS_2 flake intercepting the graphene flake is around 0.4 μm wide. Distances between the Co/TiO_2 electrodes in the reference LSV and graphene/MoS_2 LSV are 1 μm and 1.8 μm, respectively. Length of the scale bar is 1 μm. (**b**) Line profile taken from **(a)** across the MoS_2 flake along the marked line, where the thickness of the MoS_2 flake is extracted to be ~7 nm.

Supplementary Note 3

Spin signal ratio calculation

This section explains the details about the calculation for the spin signal ratio $\Delta R^{\mathrm{abs}}_{\mathrm{nl}}/\Delta R_{\mathrm{nl}}$, being $\Delta R^{\mathrm{abs}}_{\mathrm{nl}}(\Delta R_{\mathrm{nl}})$ the signal with (without) spin absorption by the MoS_2. We recall Eq. (1) from the main text for convenience[17]:

$$\frac{\Delta R_{\mathrm{nl}}^{\mathrm{abs}}}{\Delta R_{\mathrm{nl}}} = \frac{2R_{\mathrm{MoS_2}}^{\mathrm{S}}\{\sinh(L/\lambda_{\mathrm{Gr}})+2Q_{\mathrm{I}}e^{L/\lambda_{\mathrm{Gr}}}+2Q_{\mathrm{I}}^2 e^{L/\lambda_{\mathrm{Gr}}}\}}{R_{\mathrm{Gr}}^{\mathrm{S}}\{\cosh(L/\lambda_{\mathrm{Gr}})-1\}+2R_{\mathrm{MoS_2}}^{\mathrm{S}}\sinh(L/\lambda_{\mathrm{Gr}})+2R_{I}^{\mathrm{S}}\{e^{L/\lambda_{\mathrm{Gr}}}(1+Q_{\mathrm{I}})(1+2Q_{\mathrm{MoS_2}})-1\}}, \quad (11)$$

with $Q_{\mathrm{MoS_2}} = R_{\mathrm{MoS_2}}^{\mathrm{S}}/R_{\mathrm{Gr}}^{\mathrm{S}}$ and $Q_{\mathrm{I}} = R_{\mathrm{I}}^{\mathrm{S}}/R_{\mathrm{Gr}}^{\mathrm{S}}$, being $R_{\mathrm{I}}^{\mathrm{S}} = R_{\mathrm{I}}/(1-P_{\mathrm{I}}^2)$ the spin resistance of the Co/TiO_2/graphene contact and P_{I} its spin polarization. Using all the parameters mentioned in the previous sections, we calculate $\Delta R_{\mathrm{nl}}^{\mathrm{abs}}/\Delta R_{\mathrm{nl}} \approx 0.017$, predicting that the spin current traveling through the graphene channel is almost fully shunted by the MoS_2 flake in the on state of MoS_2. While Supplementary Equation 11 gives good enough qualitative understanding of the mechanism, it is assuming an approximate/simplified picture. A recent publication by Laczkowski *et al.* found the width of the absorber material is of paramount importance for the validity of Supplementary Equation 11[18]. The authors observe that, when this width becomes comparable to λ of the material where the spins propagate, Supplementary Equation 11 is not accurate anymore. This is due to the fact that Supplementary Equation 11 describes spin absorption through a point-like contact between the two materials, and therefore ignores the spin accumulation profile under the spin absorber. The authors account for this by considering an effective λ in the spin absorption area. Doing so, they calculate $\Delta R_{\mathrm{nl}}^{\mathrm{abs}}/\Delta R_{\mathrm{nl}}$ values smaller than those obtained by Supplementary Equation 11.

In our particular case, $w_{\mathrm{MoS_2}}$ is comparable to λ_{Gr}, and therefore the correction proposed by Laczkowski *et al.* should be taken into account. This leads us to $\Delta R_{\mathrm{nl}}^{\mathrm{abs}}/\Delta R_{\mathrm{nl}} < 0.017$, which further reinforces the fact that the MoS_2 acts as an extremely efficient spin absorber.

Supplementary References:

1. Tombros, N. *et al.,* Electronic spin transport and spin precession in single graphene layers at room temperature. *Nature,* **448**, 571-574 (2007).
2. Han, W. *et al*. Tunneling spin injection into single layer graphene. *Phys. Rev. Lett*. **105**, 167202 (2010).
3. Mihajlović, G., Pearson, J. E. Bader, S. D. and Hoffmann, A. Surface spin flip probability of mesoscopic Ag wires. *Phys. Rev. Lett.* **104**, 237202 (2010).
4. Idzuchi, H., Fert, A. and Otani, Y. Revisiting the measurement of the spin relaxation time in graphene-based devices. *Phys. Rev. B,* **91**, 241407(R) (2015).
5. Villamor, E., Isasa, M., Hueso, L. H. and Casanova, F. Temperature dependence of spin polarization in ferromagnetic metals using lateral spin valves. *Phys. Rev. B,* **88**, 184411 (2013).
6. Piraux, L., Dubois, S., Fert, A. and Belliard, L. The temperature dependence of the perpendicular giant magnetoresistance in Co/Cu multi-layered nanowires. *Eur. Phys. J. B*. **4**, 413 (1998).
7. Reilly, A. C. *et al.* Giant magnetoresistance of current-perpendicular exchange-biased spin-valves of Co/Cu. *IEEE Trans. Magn.* **34**, 939 (1998).
8. Song, Y. and Dery, H. Transport theory of monolayer transition-metal dichalcogenides through symmetry. *Phys. Rev. Lett*. **111**, 026601 (2013).
9. Mariani, E. and von Oppen, F. Flexural Phonons in Free-Standing Graphene. *Phys. Rev. Lett.* **100,** 076801 (2008).
10. Castro, E. V., *et al.* Limits on Charge Carrier Mobility in Suspended Graphene due to Flexural Phonons. *Phys. Rev. Lett.* **105,** 266601 (2010).

11. Jiang, J.W., Qi, Z., Park, H. S. and Rabczuk, T. Elastic bending modulus of single-layer molybdenum disulfide (MoS_2): finite thickness effect. *Nanotechnology.* **24**, 435705 (2013).
12. Kaasbjerg, K., Thygesen, K. S. and Jacobsen, K. W. Phonon-limited mobility in *n*-type single-layer MoS_2 from first principles. *Phys. Rev. B* **85**, 115317 (2012).
13. Kaasbjerg, K., Thygesen, K. S. and Jauho, A.-P. Acoustic phonon limited mobility in two-dimensional semiconductors: Deformation potential and piezoelectric scattering in monolayer MoS_2 from first principles. *Phys. Rev. B* **87,** 235312 (2013).
14. Cheiwchanchamnangij, T., Lambrecht, W. R. L., Song, Y. and Dery, H. Strain effects on the spin-orbit-induced band structure splittings in monolayer MoS_2 and graphene. *Phys. Rev. B* **88,** 155404 (2013).
15. Yang, L. *et al.* Long-lived nanosecond spin relaxation and spin coherence of electrons in monolayer MoS_2 and WS_2. *Nature Phys.* **11**, 830-834 (2015).
16. Radisavljevic, B., Radenovic, A., Brivio, J., Giacometti, V. and Kis, A. Single-layer MoS_2 transistors. *Nature Nanotech.* **6**, 147-150 (2011).
17. Niimi, Y. *et al*. Giant spin Hall induced by skew scattering from Bismuth impurities inside thin film CuBi alloys. *Phys. Rev. Lett.* **109**, 156602 (2012).
18. Laczkkowski, P. *et al.* Evaluation of spin diffusion length of AuW alloys using spin absorption experiments in the limit of large spin-orbit interactions. *Phys. Rev. B,* **92**, 214405 (2015).
